# Focus on Semaglutide 2.4 mg/week for the Treatment of Metabolic Dysfunction‐Associated Steatohepatitis

**DOI:** 10.1111/liv.70407

**Published:** 2025-10-27

**Authors:** Salvatore Petta, KyeongJin Kim, Giovanni Targher, Stefano Romeo, Silvia Sookoian, Ming‐Hua Zheng, Alessio Aghemo, Luca Valenti

**Affiliations:** ^1^ Section of Gastroenterology, PROMISE Department University of Palermo Palermo Italy; ^2^ Department of Biomedical Sciences College of Medicine Incheon Republic of Korea; ^3^ Program in Biomedical Science & Engineering College of Medicine Incheon Republic of Korea; ^4^ Research Center for Controlling Intercellular Communication (RCIC), College of Medicine Inha University Incheon Republic of Korea; ^5^ Department of Medicine University of Verona Verona Italy; ^6^ Metabolic Diseases Research Unit IRCCS Sacro Cuore ‐ Don Calabria Hospital Negrar di Valpolicella Italy; ^7^ Department of Molecular and Clinical Medicine, Institute of Medicine, Sahlgrenska Academy, Wallenberg Laboratory University of Gothenburg Gothenburg Sweden; ^8^ Department of Medicine (H7), CeRM Karolinska Institute Huddinge Sweden; ^9^ Medical Unit Endocrinology, Theme Inflammation and Ageing Karolinska University Hospital Huddinge Sweden; ^10^ Department of Cardiology Sahlgrenska University Hospital Gothenburg Sweden; ^11^ Clinical Nutrition Unit, Department of Medical and Surgical Science University Magna Graecia Catanzaro Italy; ^12^ Consejo Nacional de Investigaciones Científicas y Técnicas (CONICET) Buenos Aires Argentina; ^13^ Clinical and Molecular Hepatology, Translational Research in Health Center (CENITRES) Maimónides University Buenos Aires Argentina; ^14^ MAFLD Research Center, Department of Hepatology The First Affiliated Hospital of Wenzhou Medical University Wenzhou China; ^15^ Key Laboratory of Diagnosis and Treatment for the Development of Chronic Liver Disease in Zhejiang Province Wenzhou China; ^16^ Department of Biomedical Sciences Humanitas University Pieve Emanuele Italy; ^17^ Division of Internal Medicine and Hepatology, Department of Gastroenterology IRCCS Humanitas Research Hospital Rozzano Italy; ^18^ Department of Pathophysiology and Transplantation Università degli Studi di Milano Milan Italy; ^19^ Precision Medicine Lab, Biological Resource Center and Transfusion Medicine Fondazione IRCCS Ca Granda Ospedale Maggiore Policlinico Milan Italy

**Keywords:** fibrosis, GLP‐1, incretin, MASH, MASLD

## Abstract

Semaglutide has recently received conditional accelerated approval in the US for treatment of metabolic dysfunction‐associated steatohepatitis (MASH) with significant or advanced liver fibrosis (stage F2/F3). Phase 2 and 3 clinical trials show that subcutaneous semaglutide 2.4 mg/week leads to significant improvements in hepatic steatosis, disease activity, resolution of MASH and reduction in liver fibrosis. These benefits parallel weight loss and are accompanied by improved metabolic outcomes, including better glucose control and lipid profiles, as well as consistent benefits for cardiovascular and renal health. The treatment's safety profile is manageable, with gastrointestinal issues being the most frequent side effects, and no new safety concerns have been identified. Data on long‐term tolerability, treatment retention and clinical events are now awaited in people with MASH fibrosis. The evidence regarding semaglutide's ability to directly target the liver and improve liver damage in cirrhosis, and its impact on muscle mass in at‐risk populations, remains limited. Thus, in patients with advanced disease, it should be viewed primarily as a therapy that modifies metabolic disease. Practically, semaglutide is most suitable as a first‐line treatment to prevent liver complications for people with MASH and stage F2/F3 fibrosis with severe metabolic dysfunction, obesity, or type 2 diabetes who could benefit from both liver and cardiovascular‐renal improvements. Treatment should be tailoured to each individual, with ongoing monitoring of body weight, serum aminotransferase levels and direct measurement of liver fat and stiffness to guide therapy.

**Abbreviations**
ACC: acetyl‐CoA carboxylase
ALT: alanine aminotransferases
AMPK: AMP‐activated protein kinase
ChREBP: carbohydrate‐response element‐binding protein
ECM: extracellular matrix
ELF: enhanced liver fibrosis panel
FAS: fatty acid synthase
FAST: Fibroscan‐AST score
GLP‐1: glucagon like peptide‐1
GLP1‐R: glucagon‐like peptide‐1 receptor
GLP1‐RA: glucagon like peptide‐1 receptor agonist
HMGCR: 3‐hydroxy‐3‐methylglutaryl‐CoA reductase
HSC: hepatic stellate cells
IL‐6: interleukin‐6
LSM: liver stiffness measurement
LTA4: leukotriene A4
MACE: major adverse cardiovascular events
MASH: metabolic dysfunction‐associated steatohepatitis
MASLD: metabolic dysfunction‐associated steatotic liver disease
MCP‐1: monocyte chemoattractant protein‐1
SCD1: stearoyl‐CoA desaturase‐1
SREBP‐1c: sterol regulatory element‐binding protein 1c
T2D: type 2 diabetes
TNF‐α: tumour necrosis factor‐α
VCTE: vibration‐controlled transient elastography
α‐SMA: α‐smooth muscle actin

**Summary**

- Semaglutide has received conditional accelerated approval for treating MASH with significant or advanced liver fibrosis.
- Semaglutide improves hepatic steatosis, disease activity and favours resolution of MASH and liver fibrosis, while also promoting weight loss and enhancing metabolic health.
- The medication offers additional benefits for cardiovascular and renal health, with a manageable safety profile primarily associated with gastrointestinal side effects.
- Evidence on semaglutide's ability to directly reverse cirrhosis or impact muscle mass in at‐risk populations is limited; it is primarily considered a metabolic therapy.
- Semaglutide is best used as a first‐line treatment for MASH patients with severe metabolic dysfunction, obesity, or type 2 diabetes, with therapy tailoured and monitored individually.

## 1 Introduction

Metabolic dysfunction‐associated steatotic liver disease (MASLD) is the most common chronic liver disease worldwide, affecting up to nearly 38% of the adult population [1]. MASLD is strongly associated with type 2 diabetes (T2D) and obesity, with nearly 65%–70% of patients with T2D showing MASLD.

MASLD and metabolic dysfunction‐associated steatohepatitis (MASH), the histologic phenotype of MASLD characterised by liver injury and inflammation in addition to steatosis, is a major public health issue as it increases the risk of liver‐related complications, such as cirrhosis and hepatocellular carcinoma, while also increasing the rates of fatal and nonfatal cardiovascular events, chronic kidney disease, T2D, extrahepatic cancers [1] and severe bacterial and non‐bacterial infections [2]. Treatment for MASLD usually involves lifestyle modifications aimed at preventing cardiovascular complications, avoiding further liver damage, potentially improving fibrosis stage and, ultimately, reducing liver‐related mortality [3]. These interventions include, among others, the switch to a Mediterranean hypocaloric diet, reduced alcohol consumption and increased physical exercise [3, 4]. When focussing on liver‐related complications, the achievement of a weight loss of at least 5% is the main aim, as it is associated with improvements in liver steatosis, inflammation and fibrosis [3]. This endpoint is complex because it is difficult to achieve in clinical practice, and long‐term adherence to lifestyle interventions is questionable [5]. For these reasons, the development of effective treatments for MASLD that improve survival by reducing both hepatic and extrahepatic complications is a significant and partly unmet clinical need. The first major breakthrough in the field was the 2024 US Food and Drug Administration (FDA) conditional approval of resmetirom, an orally administered, liver‐targeted thyroid hormone receptor (THR)‐β selective drug [6]. Resmetirom, which was also approved in August 2025 by the European Medicines Agency (EMA), is indicated for the treatment of adults with non‐cirrhotic MASH with moderate to advanced fibrosis and has been administered to over 23 000 patients in the USA (https://ir.madrigalpharma.com/news‐releases/news‐release‐details/madrigal‐pharma‐reports‐second‐quarter‐2025‐financial). On August 15th, 2025, the FDA granted an accelerated and conditional approval of the subcutaneous glucagon‐like peptide (GLP‐1) receptor agonist (GLP1‐RA) semaglutide 2.4 mg/week for the treatment of MASH with moderate to advanced fibrosis (Stage F2‐F3) [7]. This represents a new indication for semaglutide, which is also approved for the treatment of T2D, prevention of cardiovascular events in people with obesity and treatment of obesity/overweight associated with metabolic disorders. The availability of semaglutide and resmetirom for the treatment of patients with MASLD/MASH with moderate and advanced fibrosis offers physicians two therapeutic options that differ in terms of mechanisms of action, safety and tolerability.

In this review article, we will focus on subcutaneous semaglutide 2.4 mg/week by analysing available efficacy and safety data to offer practical recommendations for its clinical use in people with MASLD.

## 2 Rationale for Semaglutide in MASH: Impact on Metabolism and Liver Fat

Data from the ongoing phase 3, multicentre, placebo‐controlled ESSENCE trial involving patients with biopsy‐confirmed MASH and liver fibrosis (stage F2 or F3) demonstrated that subcutaneous semaglutide 2.4 mg/week not only improves steatosis but also reduces necroinflammation, hepatocellular ballooning and fibrosis [8].

The most robust explanation for the hepatoprotective effects of semaglutide in improving and/or potentially reversing liver histological outcomes, including hepatic fat accumulation, is linked to the so‐called indirect effects of the drug. In this context, it has been proposed that semaglutide and other GLP1‐RA modulate insulin signaling pathways, thereby increasing hepatic insulin sensitivity, improving glucose uptake and utilisation and reducing hepatic gluconeogenesis [9]. Semaglutide also exerts its action on steatosis through several indirect mechanisms that contribute to substantial weight loss, including effects on caloric intake, which involve interference with central appetite suppression and satiety. Moreover, the drug reduces blood lipid levels while concomitantly modulating systemic inflammatory processes [9]. Consequently, semaglutide may beneficially affect liver steatosis by interfering with metabolic cascades and molecular pathways, including fatty acid synthesis and fatty acid β‐oxidation, which may in turn prevent the accumulation of lipid droplets in hepatocytes [9, 10, 11].

While the systemic biological effects of semaglutide that indirectly benefit MASLD/MASH are robustly validated, a knowledge gap persists concerning the drug's direct effects on the liver, alongside the scientific controversy surrounding the gene and protein expression of GLP‐1 receptor (GLP‐1R) in the liver [12]. Indeed, while several studies failed to detect GLP‐1R mRNA in the human or rodent liver [13, 14], it is conceivable that GLP‐1R might be expressed in non‐hepatic cells, such as immune cells or intrahepatic blood vessels [12]. Gupta et al. hypothesised a possible mechanism of action of GLP1‐RAs by demonstrating a protective effect on hepatocytes against death related to free fatty acids (FFAs). This would be theoretically achieved by inhibiting a dysfunctional endoplasmic reticulum stress response and reducing fatty acid accumulation through the activation of both macroautophagy and chaperone‐mediated autophagy [15]. However, it is essential to exercise caution when interpreting these results, as the study was based on the assumption that exendin‐4/GLP‐1—but not semaglutide—binds to a cognate receptor on human hepatocytes.

Therefore, is it reasonable to consider whether semaglutide directly affects liver fat or liver inflammation when GLP‐1R is not present in the liver? GLP‐1R is a 7‐transmembrane protein that functions as a receptor for the GLP‐1 hormone. The protein predominantly localises to the cellular membranes of diverse cell types throughout the human body. The most prominent molecular functions and biological processes annotated with GLP‐1R (https://functionome.geneontology.org/gene/UniProtKB:P43220) are peptide hormone binding, GLP‐1R activity, adenylate cyclase‐modulating G protein‐coupled receptor signalling pathway and positive regulation of blood pressure. This is a particularly interesting phenomenon, given that GLP‐1R is a member of the class B family of peptide hormone G protein–coupled receptors (GPCRs). When a GLP‐1RA, including semaglutide, is administered, it triggers the activation of G‐proteins, leading to an increase in the intracellular second messenger cAMP [9]. Furthermore, GLP‐1Rs, like numerous other transmembrane proteins, may initiate signalling through interactions with other proteins on the liver cell's surface or within the cell, thereby triggering a sequence of events that result in diverse cellular response processes. Besides, semaglutide might act by modifying systemic proteostasis, thereby affecting the balance between various proteins within the intrahepatic pathways of MASH. A recent study explored the circulating proteome associated with MASH in patients treated with semaglutide, identifying a ‘treatment signature’ comprising 72 unique proteins that were significantly associated with the drug dosage and MASH resolution [13]. In summary, it is evident that a considerable proportion of the pleiotropic benefits of semaglutide, including its liver‐related effects, could be attributable to still unidentified signalling pathway(s), which may be distinct from the canonical GLP‐1R activation.

## 3 Mechanisms of the Beneficial Impact of Semaglutide in Experimental Models

### 3.1 Steatosis

Semaglutide exerts potent anti‐obesity effects that contribute to the mitigation of MASLD/MASH mostly through both direct and indirect metabolic pathways. A primary mechanism involves appetite suppression and reduced caloric intake, which in turn decreases dietary lipid influx to the liver. This is particularly relevant given that approximately 15% of hepatic triglyceride (TG) content in patients with MASLD is derived directly from dietary sources. By improving systemic insulin sensitivity, semaglutide also attenuates adipose tissue lipolysis, thereby lowering the circulation of free fatty acids (FFAs) that would otherwise be delivered to the liver for TG synthesis [16]. Concomitantly, calorie restriction limits substrate supply for *de novo* lipogenesis (DNL), while semaglutide modulates lipogenic transcriptional programs governed by carbohydrate‐response element‐binding protein (ChREBP) and sterol regulatory element‐binding protein 1c (SREBP‐1c) [17], as well as their canonical downstream targets such as acetyl‐CoA carboxylase (ACC), fatty acid synthase (FAS) and stearoyl‐CoA desaturase‐1 (SCD‐1) in obese and diabetic murine models [18, 19]. Collectively, these effects converge to improve hepatic steatosis by downregulating DNL. Additional effects on nutrient handling, including delayed gastric emptying, sustained satiety and preferential reduction of central adiposity, further support metabolic re‐equilibration [20, 21]. These changes are accompanied by enhanced insulin secretion and suppression of glucagon release, reinforcing its role in metabolic homeostasis.

Notably, some preclinical data support that the benefits of semaglutide on hepatic steatosis may not be entirely dependent on weight reduction. In experimental murine models, semaglutide has been shown to modulate intracellular signaling cascades relevant to lipid synthesis. For example, the PI3K/AKT/mTORC1 pathway is a central driver of hepatic lipogenesis, activating SREBP‐1c [22]. Semaglutide has been demonstrated to suppress PI3K/AKT/mTORC1 signaling in obese mice [23], suggesting an anti‐steatotic effect independent of weight loss. Furthermore, activation of AMP‐activated protein kinase (AMPK), a master regulator of lipid and cholesterol metabolism, has been observed with semaglutide treatment, leading to downregulation of FAS, ACC and 3‐hydroxy‐3‐methylglutaryl‐CoA reductase (HMGCR) [24], while simultaneously upregulating SIRT1 activity [23]. These changes collectively reduce lipid synthesis and improve fatty acid oxidation. Additionally, semaglutide has been reported to enhance the expression of PPARα, a key transcription factor governing fatty acid uptake, activation and mitochondrial β‐oxidation [18], thereby facilitating lipid clearance from hepatocytes. Taken together, these findings indicate that the lipid‐lowering effects of semaglutide extend beyond weight control, acting through multiple molecular nodes to reduce hepatic lipid accumulation.

### 3.2 Inflammation and Oxidative Stress

Beyond its beneficial effects on lipid metabolism, semaglutide exerts meaningful anti‐inflammatory and antioxidant effects that are relevant for halting the progression from simple steatosis to MASH. In MASH‐provoking animal models, semaglutide reduced lipid peroxidation byproducts while simultaneously restoring intracellular antioxidant defenses [25], thus underscoring its ability to counteract oxidative stress. Since obesity is closely associated with a chronic pro‐inflammatory milieu, attenuation of systemic and hepatic inflammation is a crucial therapeutic target. Adipose tissue in obesity releases pro‐inflammatory cytokines such as tumor necrosis factor‐α (TNF‐α), interleukin‐6 (IL‐6) and monocyte chemoattractant protein‐1 (MCP‐1), while Kupffer cells and infiltrating macrophages in the liver amplify this inflammatory milieu, contributing to hepatocellular injury and fibrosis [26, 27].

Semaglutide treatment suppresses inflammatory signalling by downregulating mediators derived from arachidonic acid metabolism, including prostaglandin D2 and leukotriene A4 (LTA4), as well as reducing the hepatic expression of proinflammatory cytokines such as TNF‐α, IL‐1β and IL‐6 in murine MASH models [25]. In parallel, semaglutide decreases the expression of galectin‐3, which is associated with lipid accumulation and inflammation [28, 29]. These findings suggest that semaglutide not only mitigates the inflammatory response but also interferes with macrophage‐driven fibrogenic signalling [30], linking its anti‐inflammatory effects to downstream improvements in hepatic pathology.

### 3.3 Fibrosis

Preclinical studies have reported a reduction in classical fibrosis biomarkers, including α‐smooth muscle actin (α‐SMA) and genes associated with extracellular matrix (ECM) synthesis, as well as decreases in collagen deposition within the liver. These findings suggest that semaglutide may suppress *de novo* collagen production [30]. Mechanistically, semaglutide appears to downregulate TGF‐β1, a critical driver of hepatic stellate cell (HSC) activation and ECM deposition, in addition to fibrogenesis [31]. Nevertheless, it should be noted that semaglutide does not appear to accelerate the degradation or clearance of pre‐existing collagen, which limits its ability to reverse established fibrosis [32].

**FIGURE 1:**
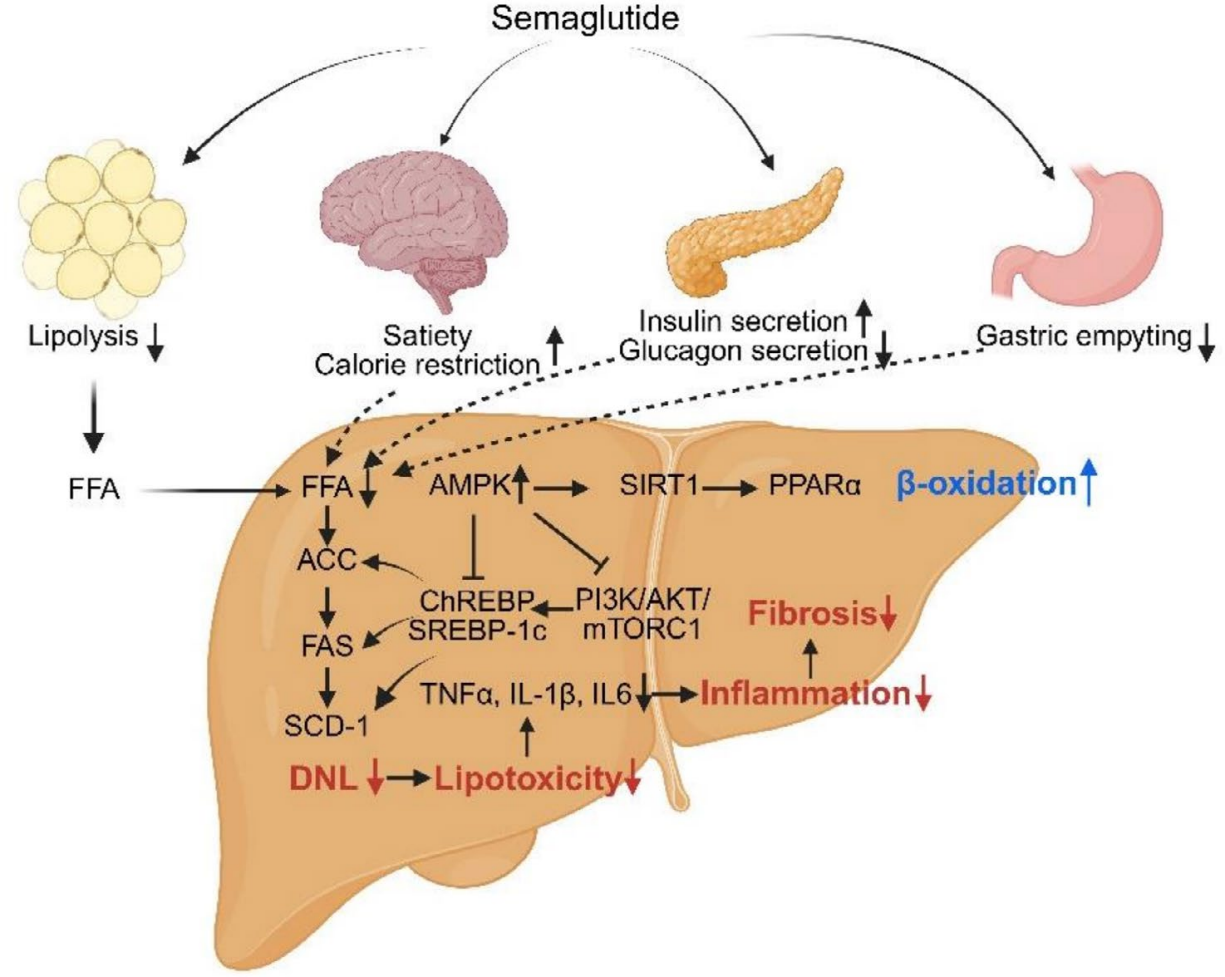
Potential mechanisms of action of Semaglutide in MASH.

Animal studies have produced mixed results, with several reporting modest anti‐fibrotic benefits, while others observed minimal improvement in fibrosis severity despite clear reductions in steatosis and inflammation. Pivotal phase 2 and 3 clinical studies have demonstrated significant reduction in steatosis, inflammation and hepatocellular ballooning, yet without corresponding improvements in fibrosis stage [32, 33]. Emerging clinical evidence, however, points to an anti‐fibrotic benefit, which may be secondary to sustained weight loss, metabolic improvement and reduced hepatic inflammation. Accordingly, while semaglutide holds promise as a disease‐modifying therapy in MASH [8, 33], its capacity to directly resolve liver fibrosis remains to be fully established. Collectively, the putative mechanisms of action of semaglutide in the liver are summarised in Figure 1.

## 4 Overview of Clinical Studies, Efficacy and Safety of Semaglutide in MASH

The clinical development of semaglutide for patients with MASH has progressed through comprehensive phase 2 and phase 3 trials, providing robust evidence across primary histological outcomes, secondary histological endpoints, non‐invasive biomarkers and metabolic parameters.

The initial phase 2 trial established the foundation for semaglutide's efficacy in MASH, enrolling 320 patients with biopsy‐confirmed NASH, the previous nomenclature for MASH and fibrosis stages F1–F3 [32]. In this 48‐week study, patients receiving subcutaneous semaglutide 0.4 mg daily achieved MASH resolution without worsening of fibrosis in 59% of cases compared to 17% in the placebo group, which was highly significant. However, improvement in fibrosis stage occurred in 43% of patients in the 0.4‐mg group versus 33% in the placebo group, failing to reach statistical significance. The mean percent weight loss was 13% in the 0.4‐mg group compared to 1% in the placebo group, demonstrating semaglutide's metabolic benefits alongside histological improvements [32].

The ESSENCE phase 3 trial represents the most substantial evidence for semaglutide's efficacy in MASH, enrolling 800 patients with biopsy‐confirmed MASH and fibrosis stage 2 or 3 [8]. Table 1 presents data on the efficacy and safety of semaglutide from the ESSENCE trial. The study demonstrated compelling results for both co‐primary endpoints at 72 weeks. Resolution of steatohepatitis without worsening of liver fibrosis was achieved in 62.9% of patients receiving semaglutide 2.4 mg weekly, compared to 34.3% in the placebo group (estimated difference, 28.7 percentage points; 95% CI, 21.1–36.2). Importantly, the phase 3 trial also demonstrated significant efficacy for the second primary endpoint: reduction in liver fibrosis without worsening of steatohepatitis occurred in 36.8% of semaglutide‐treated patients versus 22.4% of placebo recipients (estimated difference 14.4 percentage points; 95% CI 7.5–21.3). This represented a significant advancement from the phase 2 results, likely attributable to the higher dose (2.4 mg weekly vs. 0.4 mg daily), longer treatment duration and optimised study design.

**TABLE 1:** Comprehensive efficacy and safety profile of semaglutide in MASH Clinical Trials.

Parameter	Phase 3 ESSENCE (F2‐F3)
*Study characteristics*	
Dose/Regimen	2.4 mg weekly SC
Duration	72 weeks
Population	800 patients (534 sema/266 placebo)
Fibrosis stages	F2‐F3
Mean BMI (kg/m^2^)	34.6
Diabetes prevalence (%)	56.1%
F3 fibrosis (%)	68.9%
*Primary histological outcomes*	
NASH resolution without fibrosis worsening	62.9% vs. 34.3% (*p* < 0.001)
Fibrosis improvement without NASH worsening	36.8% vs. 22.4% (*p* < 0.001)
*Secondary histological outcomes*	
Combined NASH resolution + fibrosis improvement	32.7% vs. 16.1% (*p* < 0.001)
Steatosis improvement ≥ 1 grade (%)	68.8% vs. 42.5%
Ballooning improvement ≥ 1 grade (%)	74.3% vs. 53.8%
NAS reduction ≥ 2 points (%)	71.1% vs. 43.4%
Fibrosis improvement ≥ 1 stage (%)	44.6% vs. 28.6%
*Secondary non‐invasive markers*	
ELF score decrease ≥ 0.5 (%)	55.8% vs. 25.5%
Liver stiffness reduction ≥ 30% (%)	52.0% vs. 30.3%
ALT reduction ≥ 17 IU/L (%)	52.1% vs. 22.2%
AST reduction	Improved vs. placebo
FAST score improvement	Improved vs. placebo
PRO‐C3 level improvement	Improved vs. placebo
*Secondary metabolic outcomes*	
Weight loss (%)	−10.5% vs. −2.0% (*p* < 0.001)
HbA1c change ‐ diabetics (%)	−1.08% vs. 0.00%
HbA1c change—non‐diabetics (%)	−0.42% vs. +0.11%
HOMA‐IR improvement	Greater improvement in diabetics
Triglycerides change (mg/dL)	−16.8 vs. −0.3
VLDL cholesterol reduction	Improved vs. placebo
HDL cholesterol change (mg/dL)	+2.6 vs. −1.9
LDL cholesterol change (mg/dL)	−6.0 vs. −4.1
hs‐CRP reduction (mg/L)	−53.8 vs. −19.8
Systolic BP reduction (mmHg)	−5.4 vs. −1.4
*Safety outcomes*	
Any adverse event (%)	86.3% vs. 79.7%
Serious adverse events (%)	13.4% vs. 13.4%
Discontinuation due to AEs (%)	2.6% vs. 3.3%
Target dose maintenance (%)	88.0%
*Gastrointestinal events*	
Nausea (%)	36.2% vs. 13.2%
Diarrhoea (%)	26.9% vs. 12.2%
Constipation (%)	22.2% vs. 8.4%
Vomiting (%)	18.6% vs. 5.6%
*Other safety parameters*	
Acute pancreatitis (%)	0.4% vs. 0.5%
Hypoglycaemia in T2D (%)	7.4% vs. 5.4%
Deaths (*n*)	3 vs. 6

Abbreviations: AE, adverse event; BP, blood pressure; ELF, Enhanced Liver Fibrosis; FAST, FibroScan‐AST; hs‐CRP, high‐sensitivity C‐reactive protein; NAS, NAFLD Activity Score; PRO‐C3, N‐terminal propeptide of type III collagen; SC, subcutaneous; T2D, type 2 diabetes.

The ESSENCE trial revealed additional histological benefits beyond the primary endpoints. Combined resolution of steatohepatitis and reduction in liver fibrosis occurred in 32.7% of semaglutide patients versus 16.1% of placebo patients (estimated difference 16.5 percentage points; 95% CI 10.2–22.8). Analysis of individual histological components showed that more patients receiving semaglutide experienced reductions in steatosis, ballooning and total NAS. Notably, among patients with F2 fibrosis at baseline, only 8.7% of patients treated with semaglutide progressed to F3, compared to 20% of patients treated with placebo. Additionally, improvement in fibrosis stage of ≥ 1 stage was more common with semaglutide treatment (44.6% vs. 28.6% in the placebo group). These findings build upon the phase 2 results and demonstrate the enhanced efficacy achieved with the optimised dosing regimen.

Semaglutide demonstrated consistent improvements across multiple non‐invasive markers of liver health in the ESSENCE trial. The enhanced liver fibrosis (ELF) score showed favorable changes, with 55.8% of semaglutide patients achieving a clinically meaningful decrease of ≥ 0.5 compared to 25.5% of placebo patients. Liver stiffness measured by vibration‐controlled transient elastography (VCTE) revealed that 52.0% of semaglutide patients achieved ≥ 30% reduction versus 30.3% of placebo recipients. Liver enzymes showed early and sustained improvements, with alanine aminotransferase (ALT) and aspartate aminotransferase (AST) levels declining significantly by 12 weeks and maintaining improvement throughout the study period. The percentage of patients achieving ALT reduction ≥ 17 IU/L was 52.1% with semaglutide versus 22.2% with placebo. The FibroScan‐AST (FAST) score and plasma N‐terminal propeptide of type III collagen (PRO‐C3) levels also improved with semaglutide treatment, providing objective biomarker evidence to support the histological findings [8].

Weight reduction represents one of semaglutide's most consistent benefits across all studies. In the ESSENCE trial, at week 72, patients achieved a mean weight reduction of 10.5% with semaglutide versus 2.0% with placebo (estimated difference −8.5 percentage points). This was comparable to the 13% weight loss observed in the phase 2 trial with the 0.4 mg daily dose, indicating consistent metabolic efficacy across different dosing regimens. Glycaemic control improved substantially in patients with T2D. In the ESSENCE trial, patients with T2D showed HbA1c reduction of −1.08% with semaglutide versus 0.0% with placebo, while those without T2D showed a reduction of −0.42% versus +0.11% with placebo. Insulin resistance, as estimated by HOMA‐IR, showed greater improvement in patients with T2D compared to those without T2D. Lipid profiles also showed consistent improvements, particularly in circulating levels of triglycerides and very low‐density lipoprotein (VLDL) cholesterol. The ESSENCE trial reported significant reductions in plasma triglycerides (mean absolute change −16.77 mg/dL vs. −0.27 mg/dL with placebo) and improvements in plasma HDL cholesterol (+2.62 mg/dL vs. −1.95 mg/dL). Plasma high‐sensitivity C‐reactive protein levels decreased significantly by 53.8 mg/L with semaglutide versus 19.8 mg/L with placebo, indicating reduced systemic chronic inflammation. Blood pressure also improved, with systolic pressure reducing by 5.4 mmHg with semaglutide versus 1.4 mmHg with placebo.

Semaglutide demonstrated a favourable safety profile consistent with its established use in T2D and obesity across both phase 2 and phase 3 studies. In the ESSENCE trial, 86.3% of patients treated with semaglutide experienced adverse events, compared to 79.7% of patients receiving placebo, with serious adverse events occurring equally in both treatment arms (13.4%). Importantly, only 2.6% of patients treated with semaglutide discontinued treatment due to adverse events, compared to 3.3% in the placebo group. Gastrointestinal disorders represented the most common adverse events, including nausea (36.2% vs. 13.2%), diarrhoea (26.9% vs. 12.2%), constipation (22.2% vs. 8.4%) and vomiting (18.6% vs. 5.6%). These events were typically mild to moderate and transient, occurring primarily during dose escalation. The incidence of acute pancreatitis was similar between groups (0.4% vs. 0.5%). Hypoglycaemia occurred in 7.4% of semaglutide patients with type 2 diabetes versus 5.4% with placebo, with only level 2 or 3 events counted. Target dose maintenance was excellent, with 88.0% of patients maintaining the target dose of 2.4 mg until week 72. Nine patients died during the study (three in the semaglutide group and six in the placebo group), with no evident clustering of cause of death. The safety profile remained consistent with previous semaglutide studies, with no new safety signals identified. However, no data were reported about the impact of semaglutide treatment on muscle mass loss, this finding being worthy of further exploration in a population at high risk of sarcopenic obesity, like MASH patients with fibrosis [34].

The comprehensive clinical evidence from phase 2 and phase 3 trials supports semaglutide as an effective therapy for MASH with fibrosis stages F2–F3, showing significant histological improvements alongside substantial metabolic benefits and an acceptable safety profile.

## 5 Semaglutide in Cirrhosis

The efficacy of semaglutide in patients with MASH‐related compensated cirrhosis has formally been assessed only in one phase 2 RCT [33]. In this small trial, 71 patients with biopsy‐proven compensated cirrhosis and BMI ≥ 27 kg/m^2^ (75% with T2D, mean BMI 34.9 kg/m^2^) were randomised to semaglutide 2.4 mg once weekly (*n* = 47) or placebo (*n* = 24) for 48 weeks. Semaglutide conferred the expected metabolic benefits, including significant weight loss, improved glycaemic control and reductions in liver fat and inflammatory markers. Safety was acceptable, with no hepatic decompensation events or deaths reported, and both liver and kidney function remained stable. However, the trial did not meet its primary endpoint. Fibrosis regression without worsening of MASH was observed in 11% of semaglutide‐treated patients compared with 29% in the placebo group (OR 0.28, 95% CI 0.06–1.24; *p* = 0.09). Similarly, rates of MASH resolution did not differ significantly between the groups. Notably, the lack of supportive signals from non‐invasive fibrosis measures, including magnetic resonance elastography, suggests that the seemingly greater fibrosis improvement in the placebo group may have been affected by biopsy variability and the unmasking effect of reduced steatosis [35].

These results highlight a key concept: while semaglutide improves the metabolic milieu that drives disease progression, it does not seem to reverse established cirrhosis within the studied timeframe. This aligns with the lack of direct GLP‐1 receptor signalling in human hepatocytes [36], possibly leading to an ‘irreversibility threshold’ on the beneficial effect on liver damage once advanced architectural remodelling has occurred. On the other hand, different drug classes, namely fibroblast growth factor receptor‐21 (FGF‐21) analogues, that target directly hepatocytes, but also adipose tissue and the central nervous system, and possibly also hepatic stellate cells [37, 38, 39], have shown a potential to reverse early‐stage cirrhosis [40]. From a clinical perspective, semaglutide in cirrhotic patients should therefore be considered primarily a metabolic intervention. It is useful for controlling weight, glycaemia and cardiovascular risk, but it does not function as an antifibrotic therapy. Two complementary strategies may help to overcome this limitation. First, GLP‐1RAs should be introduced earlier in the disease course, particularly in patients with significant but not yet cirrhotic fibrosis (stages F1–F3), where they have demonstrated the ability to slow progression and induce MASH resolution. Second, combining GLP‐1RAs with liver‐directed therapies, such as resmetirom, might provide synergistic benefits by addressing both the upstream metabolic drivers and the intrahepatic fibrosis processes of MASH [41, 42]. Importantly, although semaglutide appeared safe in this specific patient population, additional larger and longer‐term studies, including also people with more severe liver disease, will be necessary to examine not only the potential benefits, but also the safety of semaglutide in patients with a hypercatabolic state at high risk of sarcopenia, which has a detrimental impact on prognosis [43]. However, the relatively short study duration (only 48 weeks, including the drug titration phase) and the small sample size do not allow for a definitive conclusion regarding the potential efficacy and safety of semaglutide in patients with MASH‐related cirrhosis.

## 6 Impact of Semaglutide on Cardiovascular and Renal Outcomes

Substantial evidence from large multicentre, placebo‐controlled RCTs has shown that treatment with once‐weekly subcutaneous semaglutide (or other GLP‐1RAs) reduces the risk of adverse cardiovascular and renal outcomes, as well as hospitalisations for heart failure in patients with overweight or obesity, regardless of their T2D status [44].

In a 2021 meta‐analysis of eight large phase 3 cardiovascular outcome RCTs, involving 60 080 patients with T2D, Sattar et al. reported that GLP‐1RAs, regardless of structural homology, reduced the risk of major adverse cardiovascular events (i.e., 3‐point major adverse cardiovascular events (MACE), including nonfatal myocardial infarction, nonfatal stroke, or cardiovascular death) by 14% (HR 0.86, 95% CI 0.80–0.93), all‐cause mortality by 12% (HR 0.88, 95% CI 0.82–0.94) and hospital admissions for heart failure by 11% (HR 0.89, 95% CI 0.82–0.98) [45]. In another meta‐analysis of five cardiovascular outcome RCTs, Barkas et al. reported that compared to placebo, GLP‐1RAs significantly reduced the risk of developing ischemic stroke by 13% (HR 0.87; 95% CI 0.78–0.98) [46].

Interestingly, the cardiovascular benefits of GLP‐1RAs do not seem to be limited only to patients with T2D [47, 48]. In the SELECT trial, an event‐driven superiority phase 3 placebo‐controlled RCT that included 17 604 participants with preexisting cardiovascular disease and overweight/obesity but no history of T2D, Lincoff et al. reported that semaglutide 2.4 mg/week was superior to placebo in reducing the risk of 3‐point MACEs over a mean follow‐up of ~3.5 years (HR 0.80; 95% CI 0.72–0.90) [47]. A subgroup analysis of the SELECT trial showed that semaglutide 2.4 mg/week was effective in lowering the risk of MACEs by 21% and 36% in patients with an intermediate and high likelihood of advanced liver fibrosis, as identified by a baseline FIB‐4 index of ≥ 1.3 and ≥ 2.67, respectively. This suggests that the cardiovascular benefits of semaglutide also extend to a patient population with this liver condition [49]. Recently, in a post hoc pooled participant‐level analysis of four placebo‐controlled RCTs, Kosiborod et al. examined the effects of once‐weekly subcutaneous semaglutide (2.4 mg/week in SELECT, STEP‐HFpEF and STEP‐HFpEF DM; 1.0 mg/week in FLOW) on heart failure events. These investigators found that obese patients who had heart failure with preserved ejection fraction, semaglutide significantly reduced the risk of the combined endpoint of cardiovascular death or worsening heart failure events (HR 0.69, 95% CI 0.53–0.89), and worsening heart failure events alone (HR 0.59, 95% CI 0.41–0.82) [50].

Data on the renal benefits of GLP‐1RAs were initially reported from analyses of secondary outcomes in cardiovascular outcome trials, which also measured estimated glomerular filtration rate (eGFR) and albuminuria [44]. In a 2021 meta‐analysis of cardiovascular outcome RCTs published by Sattar et al., GLP‐1RAs significantly reduced the risk of a composite kidney outcome consisting of the development of abnormal albuminuria, doubling of serum creatinine, or at least a 40% decline in eGFR, kidney replacement therapy, or kidney‐related death by 21% (HR 0.79; 95% CI 0.73–0.87) [45]. Recently, the phase 3 placebo‐controlled FLOW (Evaluate Renal Function with Semaglutide Once Weekly) trial showed that once‐weekly subcutaneous semaglutide (at a dose of 1.0 mg) for a median of 3.4 years was superior to placebo in reducing the risk of major kidney disease events (HR 0.79; 95% CI 0.66–0.94), i.e., a composite of the onset of kidney failure, at least a 50% reduction in eGFR from baseline, or death from kidney‐related causes, in 3533 patients with T2D and chronic kidney disease [51]. Interestingly, in a subsequent analysis of the same FLOW trial, the investigators also showed that the benefits of semaglutide in improving major kidney outcomes were consistent in participants with or without concomitant use of SGLT2 inhibitors [52].

## 7 Predictors of Response

In real‐world analyses of 7847 Italian individuals with T2D on GLP‐1RAs, women had a 16% higher probability of discontinuation, with no differences in dose escalation or maximum dose [53]. Over more than 5 years of follow‐up, women showed ~1 kg greater weight loss but no difference in HbA1c reduction. Given their higher dropout rate, the population‐level benefit might be attenuated. Theoretically, since body weight is a key determinant of MASLD, even a 1‐kg difference could matter in the long term, but whether this statistically significant difference translates into clinical benefits remains uncertain. A meta‐analysis of RCTs reported no strong predictors of HbA1c response, except for lower baseline insulin production [54]. Ethnicity showed a moderate interaction: people of African or Asian ancestry experienced greater cardiovascular benefit compared to those of European ancestry after GLP‐1RAs agonist treatment [54].

MASLD is highly heritable [55]. Common *PNPLA3* and *TM6SF2* variants strongly influence onset and interact with excess body weight, amplifying liver injury. GLP‐1RAs may, therefore, have greater benefit in carriers of these variants. This is consistent with data showing larger serum ALT reductions in *PNPLA3* p.I148M (rs738409) carriers treated with semaglutide [56]. The impact of GLP‐1RAs on liver‐related events according to the genetic background remains, however, to be clarified.

Recent studies suggest at least two MASLD subtypes: one driven by lipoprotein retention and the other by increased DNL with impaired β‐oxidation [57]. Whether GLP‐1 RAs agonists may have different effects on these mechanistic subtypes remains an open question. Alcohol intake may also influence response, as GLP‐1 RAs agonists reduce alcohol consumption [58], potentially offering dual benefits for liver disease.

Sub‐group analyses from the phase 3 ESSENCE trial also reported that the effects of semaglutide on both MASH resolution and fibrosis improvement remain consistent across age, sex, presence of T2D and BMI categories. However, the difference versus placebo was not significant in patients with BMI < 27 kg/m^2^ for fibrosis improvement. These data should be interpreted with caution due to post hoc analyses on sometimes small subgroups and are therefore worthy of confirmation and further exploration in real‐life studies.

Overall, it is still early to identify predictors of MASLD response to GLP‐1RAs. Future research should consider the genetic background, metabolic subtypes, sex and lifestyle factors to discover reliable predictors, especially for liver‐related events in MASLD people treated with semaglutide. 

## 8 Who and When to Treat?

According to regulatory agencies and the recommendations of scientific societies [3, 42], semaglutide is now an option as a first‐line treatment for people with MASH and fibrosis stage F2–F3, irrespective of the severity of metabolic comorbidities and the presence of obesity and T2D [59]. Thanks to the pleiotropic effects on glucose control and weight loss, and the ability to prevent cardiovascular events in people with severe metabolic dysfunction, the ideal candidates that are most likely to benefit, both concerning hepatic and cardiometabolic outcomes, are patients with severe overweight/obesity and/or T2D [42]. Indeed, it is important to note that in people with MASH and fibrosis stage F2–F3, cardiovascular disease remains the leading cause of morbidity and mortality [60]. However, the benefit of switching to semaglutide in patients who are already on other GLP‐1RA or dual incretin agonists [61] is currently unproven. Combination with resmetirom, which has a different, liver‐directed mechanism of action aimed at reducing hepatic fat, will likely be the more reasonable option for patients on GLP‐1RA with persistent MASH and fibrosis. In our opinion, semaglutide and GLP‐1RA should not be discontinued due to the protective effect on cardiometabolic and renal comorbidities.

Current evidence does not support the prescription of semaglutide to induce liver fibrosis regression in patients with MASLD and cirrhosis [33]. However, semaglutide can be used to treat T2D and obesity, with caution regarding its potential detrimental effects on sarcopenia [43]. Combination with nutritional supplements [62] or physical activity programs [63] may represent an option in this setting.

Finally, one could argue that in the long term, the benefit of semaglutide might be even greater in younger patients at an earlier stage of disease with less severe liver fibrosis, to prevent the progression to significant liver disease, CVD and renal complications. Additional studies are necessary to test this hypothesis and to prove its cost‐effectiveness. Meanwhile, semaglutide can be prescribed to all patients who have other indications, with a high likelihood of reducing liver fat accumulation and the progression to hepatic and extra‐hepatic diseases [64].

**FIGURE 2:**
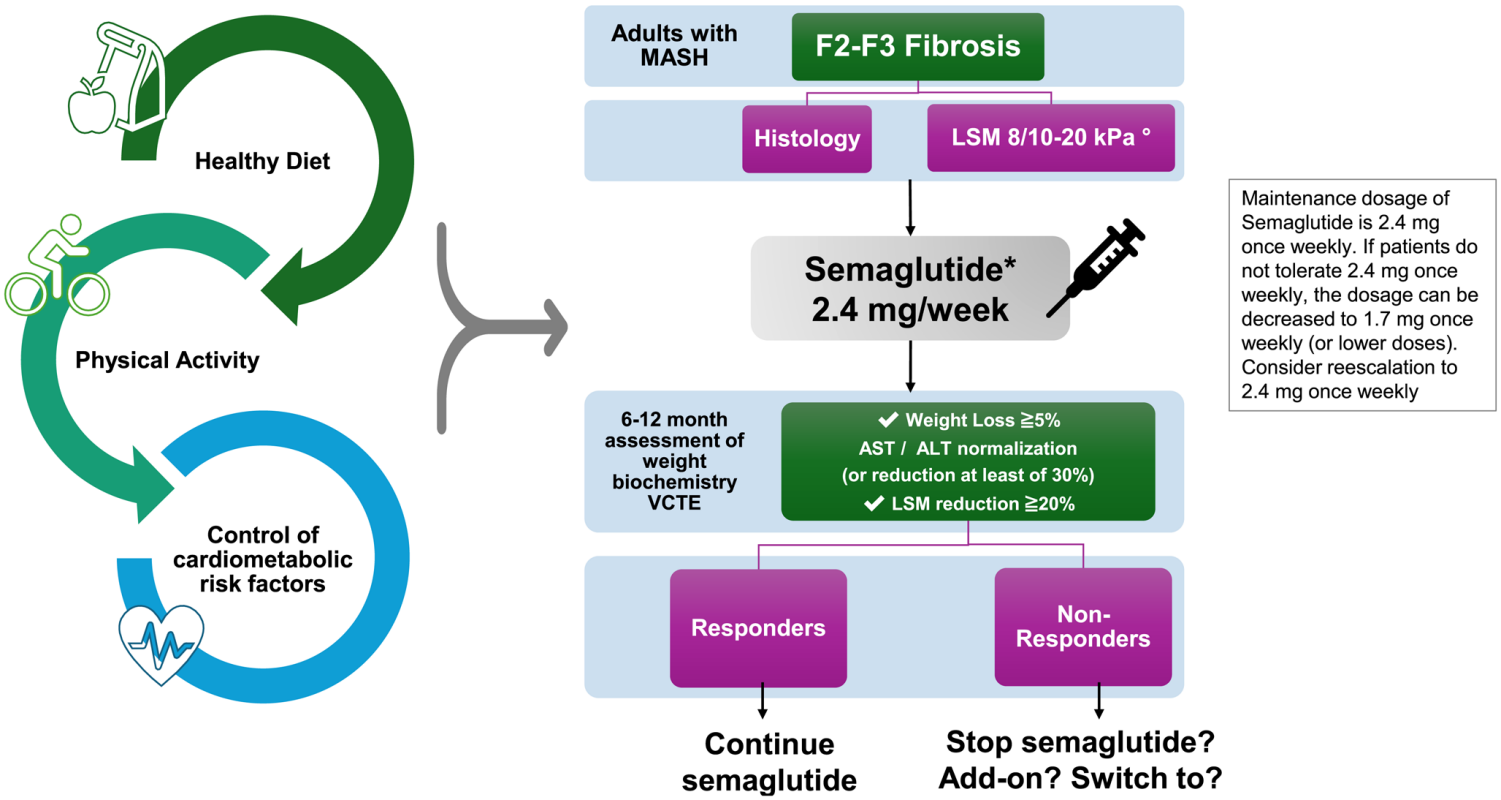
Potential algorithm to manage Semaglutide treatment for adult patients with MASH in clinical practice. LSM: liver stiffness measurement; VCTE: vibration controlled transient elastography. Please note that LSM cannot accurately discriminate between histological F2/F3 fibrosis. *In people who are not already on GLP1‐RAs, and in particular in the presence of T2D and/or obesity. Gallstone disease and family history of medullary thyroid cancer are contraindications. Resmetirom may be an alternative; °LSM threshold recommended to consider treatment remains to be defined.

Coming back to patients with MASH, the selection of those with F2–F3 fibrosis can be based on non‐invasive criteria, such as fibrosis biomarker panels or, most commonly, increased liver stiffness measurement (LSM) [6, 65]. Evidence consistently demonstrates that LSM below 8 kPa can effectively rule out patients at risk for advanced fibrosis, whereas values at or above 10 kPa can efficiently detect compensated advanced chronic liver disease with increased risk for long‐term liver‐related complications [66]. Furthermore, the predictive capacity of Fibroscan‐derived LSM for liver‐related events equals or exceeds that of histological at‐risk MASH or histological fibrosis assessment [67]. Accordingly, LSM serves as an effective surrogate marker for determining patients who would benefit from semaglutide therapy, with the 10 kPa LSM cutoff likely identifying those requiring the most immediate clinical intervention, while emphasizing the importance of excluding individuals with LSM exceeding 20 kPa or those presenting clinical, imaging, or endoscopic evidence of portal hypertension, circumstances where semaglutide prescription is currently not specifically indicated. The clinical availability of semaglutide raises another critical consideration: establishing how and when to assess treatment response. Clinical trial evidence suggests that histological responders can be identified by a minimum 30% reduction in MRI‐PDFF‐measured hepatic fat content and a decrease of at least 17 IU in serum ALT levels from baseline values at 3 months [68]. Large cohort studies have shown that a reduction of at least 20% in LSM during follow‐up predicts a decrease in the risk of developing long‐term liver‐related events [69]. Additionally, research supports the notion that lifestyle‐based interventions resulting in a weight reduction of at least 5% over one year can produce histological steatosis improvement. In contrast, weight loss of 7%–10% or greater may achieve MASH resolution and reduce fibrosis [70]. Given the substantial impact of semaglutide on weight reduction, this parameter could serve as a response indicator. Proposed evaluation strategies could encompass monitoring weight loss of a minimum of 5%, a serum ALT level decrease and a 20% LSM reduction at 6–12 months intervals. In case of lack of surrogate response or in case of clinical progression, the choice to stop the treatment or add another drug should be based on the liver and metabolic risk profiles of the individual patient. Figure 2 illustrates a possible algorithm for managing semaglutide treatment in clinical practice.

## 9 Conclusions

Semaglutide has become a major pharmacologic advance for patients with MASLD/MASH. Robust phase 3 data show that subcutaneous semaglutide (2.4 mg weekly) leads to clinically meaningful and consistent improvements in hepatic steatosis, necroinflammation, fibrosis and resolution of MASH, accompanied by substantial weight loss and positive metabolic effects (better glucose control, lipids and blood pressure), as well as consistent cardiovascular and renal benefits. The safety profile of semaglutide in controlled trials is predictable and manageable, with gastrointestinal effects being the most common adverse events, and no new safety signals have been identified so far.

Evidence that semaglutide benefits patients with established cirrhosis through cirrhosis regression or a reduction in liver‐related complications is modest or inconsistent; therefore, in this clinical setting, semaglutide should, therefore, be regarded primarily as a metabolic disease‐modifying therapy rather than a proven antifibrotic capable of reversing advanced scarring. Uncertainties also persist about the drug's direct hepatic mechanisms (given unresolved questions about intrahepatic GLP‐1 receptor expression), the long‐term durability of histological responses, effects on muscle mass in elderly populations at risk for sarcopenic obesity, and the optimal patient selection strategy in real‐world settings.

In addition, the potential beneficial impact of semaglutide in reducing alcohol intake in people with MASH and harmful consumption [71, 72], especially in those with MetALD, should be evaluated in further studies.

From a practical standpoint, semaglutide is best positioned for patients with active MASH and clinically significant fibrosis (particularly F2–F3 by biopsy or concordant non‐invasive testing), especially those with obesity and/or T2D who stand to gain both hepatic and cardio‐renal benefits. Treatment management decisions should be individualised, with baseline and on‐treatment monitoring using weight, ALT (an early biochemical change), MRI‐PDFF, or a ≥ 30% PDFF change, where available, and longitudinal LSM (targeting a ≥ 20% reduction) guiding clinical decisions.

Further studies are needed to examine the clinical benefit of semaglutide in people with less severe liver fibrosis, as well as its combination with resmetirom or other pharmacological approaches in those with aggressive or treatment‐resistant MASH.

## Author Contributions

All authors contributed to drafting the manuscript and approved the last version.

## Conflicts of Interest

L.V. reports speaking fees from: Viatris, Novo Nordisk, GSK; consulting for: Novo Nordisk, Pfizer, Boehringer Ingelheim, Resalis, GSK, ALMAC, AIRNA. S.P. served as a speaker or advisor for Boehringer, Echosens, Madrigal, MSD, Novo Nordisk, Pfizer and Resalis. A.A. served as a speaker or advisor for Madrigal, MSD, Novo Nordisk, AbbVie, Gilead Sciences, Ipsen and BMS. Other authors do not declare any relevant conflict of interest.

## Data Availability

Data sharing not applicable to this article as no datasets were generated or analysed during the current study.

## References

[1] G. Targher , L. Valenti , and C. D. Byrne , “Metabolic Dysfunction‐Associated Steatotic Liver Disease,” New England Journal of Medicine 393, no. 7 (2025): 683–698. 40802944 10.1056/NEJMra2412865

[2] G. Targher , H. Tilg , and L. Valenti , “Risk of Serious Bacterial and Non‐Bacterial Infections in People With MASLD,” Liver International 45, no. 4 (2025): e70059. 40072231 10.1111/liv.70059 PMC11899495

[3] European Association for the Study of the Liver , European Association for the Study of Diabetes , and European Association for the Study of Obesity , “EASL‐EASD‐EASO Clinical Practice Guidelines on the Management of Metabolic Dysfunction‐Associated Steatotic Liver Disease (MASLD),” Journal of Hepatology 81, no. 3 (2024): 492–542. 38851997 10.1016/j.jhep.2024.04.031

[4] I. Alabdul Razzak , A. Fares , J. G. Stine , and H. D. Trivedi , “The Role of Exercise in Steatotic Liver Diseases: An Updated Perspective,” Liver International 45, no. 1 (2025): e16220. 39720849 10.1111/liv.16220 PMC12536350

[5] N. Pugliese , I. Arcari , F. Cerini , et al., “Key Predictors of Relevant Weight Loss in Patients With Metabolic Dysfunction‐Associated Steatotic Liver Disease,” United European Gastroenterology Journal 13 (2025): 1205–1216. 40468530 10.1002/ueg2.70055 PMC12463688

[6] S. Petta , G. Targher , S. Romeo , et al., “The First MASH Drug Therapy on the Horizon: Current Perspectives of Resmetirom,” Liver International 44, no. 7 (2024): 1526–1536. 38578141 10.1111/liv.15930

[7] U.S. Food and Drug Administration , ‘FDA Approves Treatment for Serious Liver Disease Known as ‘MASH’ (2025).

[8] A. J. Sanyal , P. N. Newsome , I. Kliers , et al., “Phase 3 Trial of Semaglutide in Metabolic Dysfunction‐Associated Steatohepatitis,” New England Journal of Medicine 392, no. 21 (2025): 2089–2099. 40305708 10.1056/NEJMoa2413258

[9] Z. Zheng , Y. Zong , Y. Ma , et al., “Glucagon‐Like Peptide‐1 Receptor: Mechanisms and Advances in Therapy,” Signal Transduction and Targeted Therapy 9, no. 1 (2024): 234. 39289339 10.1038/s41392-024-01931-z PMC11408715

[10] M. J. Armstrong , D. Hull , K. Guo , et al., “Glucagon‐Like Peptide 1 Decreases Lipotoxicity in Non‐Alcoholic Steatohepatitis,” Journal of Hepatology 64, no. 2 (2016): 399–408. 26394161 10.1016/j.jhep.2015.08.038 PMC4713865

[11] G. Svegliati‐Baroni , S. Saccomanno , C. Rychlicki , et al., “Glucagon‐Like Peptide‐1 Receptor Activation Stimulates Hepatic Lipid Oxidation and Restores Hepatic Signalling Alteration Induced by a High‐Fat Diet in Nonalcoholic Steatohepatitis,” Liver International 31, no. 9 (2011): 1285–1297. 21745271 10.1111/j.1478-3231.2011.02462.x

[12] B. A. Mclean , C. K. Wong , J. E. Campbell , D. J. Hodson , S. Trapp , and D. J. Drucker , “Revisiting the Complexity of GLP‐1 Action From Sites of Synthesis to Receptor Activation,” Endocrine Reviews 42, no. 2 (2021): 101–132. 33320179 10.1210/endrev/bnaa032 PMC7958144

[13] M. Jara , J. Norlin , M. S. Kjaer , et al., “Modulation of Metabolic, Inflammatory and Fibrotic Pathways by Semaglutide in Metabolic Dysfunction‐Associated Steatohepatitis,” Nature Medicine 31 (2025): 3128–3140. 10.1038/s41591-025-03799-0 PMC12443624 40691365

[14] N. Panjwani , E. E. Mulvihill , C. Longuet , et al., “GLP‐1 Receptor Activation Indirectly Reduces Hepatic Lipid Accumulation but Does Not Attenuate Development of Atherosclerosis in Diabetic Male ApoE(−/−) Mice,” Endocrinology 154, no. 1 (2013): 127–139. 23183176 10.1210/en.2012-1937

[15] N. A. Gupta , J. Mells , R. M. Dunham , et al., “Glucagon‐Like Peptide‐1 Receptor Is Present on Human Hepatocytes and Has a Direct Role in Decreasing Hepatic Steatosis In Vitro by Modulating Elements of the Insulin Signaling Pathway,” Hepatology 51, no. 5 (2010): 1584–1592. 20225248 10.1002/hep.23569 PMC2862093

[16] S. Bandyopadhyay , S. Das , S. S. Samajdar , and S. R. Joshi , “Role of Semaglutide in the Treatment of Nonalcoholic Fatty Liver Disease or Non‐Alcoholic Steatohepatitis: A Systematic Review and Meta‐Analysis,” Diabetes & Metabolic Syndrome 17, no. 10 (2023): 102849. 37717295 10.1016/j.dsx.2023.102849

[17] T. Dusilova , J. Kovar , I. Lankova , et al., “Semaglutide Treatment Effects on Liver Fat Content in Obese Subjects With Metabolic‐Associated Steatotic Liver Disease (MASLD),” Journal of Clinical Medicine 13, no. 20 (2024): 6100. 39458050 10.3390/jcm13206100 PMC11508983

[18] R. M. Pontes‐Da‐Silva , T. De Souza Marinho , L. E. De Macedo Cardoso , C. A. Mandarim‐De‐Lacerda , and M. B. Aguila , “Obese Mice Weight Loss Role on Nonalcoholic Fatty Liver Disease and Endoplasmic Reticulum Stress Treated by a GLP‐1 Receptor Agonist,” International Journal of Obesity (London, England: 2001) 46, no. 1 (2022): 21–29. 10.1038/s41366-021-00955-7 34465857

[19] M. Soto‐Catalan , L. Opazo‐Rios , H. Quiceno , et al., “Semaglutide Improves Liver Steatosis and De Novo Lipogenesis Markers in Obese and Type‐2‐Diabetic Mice With Metabolic‐Dysfunction‐Associated Steatotic Liver Disease,” International Journal of Molecular Sciences 25, no. 5 (2024): 2961. 38474208 10.3390/ijms25052961 PMC10932050

[20] D. J. Drucker , “Mechanisms of Action and Therapeutic Application of Glucagon‐Like Peptide‐1,” Cell Metabolism 27, no. 4 (2018): 740–756. 29617641 10.1016/j.cmet.2018.03.001

[21] X. C. Wang , A. M. Gusdon , H. Liu , and S. Qu , “Effects of Glucagon‐Like Peptide‐1 Receptor Agonists on Non‐Alcoholic Fatty Liver Disease and Inflammation,” World Journal of Gastroenterology 20, no. 40 (2014): 14821–14830. 25356042 10.3748/wjg.v20.i40.14821 PMC4209545

[22] J. L. Yecies , H. H. Zhang , S. Menon , et al., “Akt Stimulates Hepatic SREBP1c and Lipogenesis Through Parallel mTORC1‐Dependent and Independent Pathways,” Cell Metabolism 14, no. 1 (2011): 21–32. 21723501 10.1016/j.cmet.2011.06.002 PMC3652544

[23] P. H. Reis‐Barbosa , I. A. Marcondes‐De‐Castro , T. S. Marinho , M. B. Aguila , and C. A. Mandarim‐De‐Lacerda , “The mTORC1/AMPK Pathway Plays a Role in the Beneficial Effects of Semaglutide (GLP‐1 Receptor Agonist) on the Liver of Obese Mice,” Clinics and Research in Hepatology and Gastroenterology 46, no. 6 (2022): 101922. 35427802 10.1016/j.clinre.2022.101922

[24] C. Fang , J. Pan , N. Qu , et al., “The AMPK Pathway in Fatty Liver Disease,” Frontiers in Physiology 13 (2022): 970292. 36203933 10.3389/fphys.2022.970292 PMC9531345

[25] S. Niu , S. Chen , X. Chen , et al., “Semaglutide Ameliorates Metabolism and Hepatic Outcomes in an NAFLD Mouse Model,” Frontiers in Endocrinology 13 (2022): 1046130. 36568109 10.3389/fendo.2022.1046130 PMC9780435

[26] D. Ezhilarasan , “Oxidative Stress is Bane in Chronic Liver Diseases: Clinical and Experimental Perspective,” Arab Journal of Gastroenterology 19, no. 2 (2018): 56–64. 29853428 10.1016/j.ajg.2018.03.002

[27] S. L. Friedman , B. A. Neuschwander‐Tetri , M. Rinella , and A. J. Sanyal , “Mechanisms of NAFLD Development and Therapeutic Strategies,” Nature Medicine 24, no. 7 (2018): 908–922. 10.1038/s41591-018-0104-9 PMC6553468 29967350

[28] L. V. Herrera‐Marcos , R. Martinez‐Beamonte , M. Macias‐Herranz , et al., “Hepatic Galectin‐3 Is Associated With Lipid Droplet Area in Non‐Alcoholic Steatohepatitis in a New Swine Model,” Scientific Reports 12, no. 1 (2022): 1024. 35046474 10.1038/s41598-022-04971-z PMC8770509

[29] D. Ezhilarasan , “Unraveling the Pathophysiologic Role of Galectin‐3 in Chronically Injured Liver,” Journal of Cellular Physiology 238, no. 4 (2023): 673–686. 36745560 10.1002/jcp.30956

[30] M. B. Mollerhoj , S. S. Veidal , K. T. Thrane , et al., “Hepatoprotective Effects of Semaglutide, Lanifibranor and Dietary Intervention in the GAN Diet‐Induced Obese and Biopsy‐Confirmed Mouse Model of NASH,” Clinical and Translational Science 15, no. 5 (2022): 1167–1186. 35143711 10.1111/cts.13235 PMC9099137

[31] J. A. Inia , G. Stokman , M. C. Morrison , et al., “Semaglutide Has Beneficial Effects on Non‐Alcoholic Steatohepatitis in Ldlr−/− Leiden Mice,” International Journal of Molecular Sciences 24, no. 10 (2023): 8494. 37239841 10.3390/ijms24108494 PMC10218334

[32] P. N. Newsome , K. Buchholtz , K. Cusi , et al., “A Placebo‐Controlled Trial of Subcutaneous Semaglutide in Nonalcoholic Steatohepatitis,” New England Journal of Medicine 384, no. 12 (2021): 1113–1124. 33185364 10.1056/NEJMoa2028395

[33] R. Loomba , M. F. Abdelmalek , M. J. Armstrong , et al., “Semaglutide 2.4 Mg Once Weekly in Patients With Non‐Alcoholic Steatohepatitis‐Related Cirrhosis: A Randomised, Placebo‐Controlled Phase 2 Trial,” Lancet Gastroenterology & Hepatology 8, no. 6 (2023): 511–522. 36934740 10.1016/S2468-1253(23)00068-7 PMC10792518

[34] Y. H. Lee , K. S. Jung , S. U. Kim , et al., “Sarcopaenia Is Associated With NAFLD Independently of Obesity and Insulin Resistance: Nationwide Surveys (KNHANES 2008‐2011),” Journal of Hepatology 63, no. 2 (2015): 486–493. 25772036 10.1016/j.jhep.2015.02.051

[35] A. Bhateja , R. Sharma , and A. Chauhan , “Semaglutide in NASH‐Related Cirrhosis: Still a Long Way to Go?,” Lancet Gastroenterology & Hepatology 8, no. 8 (2023): 694. 10.1016/S2468-1253(23)00109-7 37453428

[36] F. Bril , “Semaglutide in NASH‐Related Cirrhosis: Too Late to the Party?,” Lancet Gastroenterology & Hepatology 8, no. 6 (2023): 494–495. 36934739 10.1016/S2468-1253(23)00069-9

[37] J. P. Rose , D. A. Morgan , A. I. Sullivan , et al., “FGF21 Reverses MASH Through Coordinated Actions on the CNS and Liver,” Cell Metabolism 37, no. 7 (2025): 1515–1529.e6. 40367940 10.1016/j.cmet.2025.04.014 PMC12409791

[38] S. A. Harrison , T. Rolph , M. Knott , and J. Dubourg , “FGF21 Agonists: An Emerging Therapeutic for Metabolic Dysfunction‐Associated Steatohepatitis and Beyond,” Journal of Hepatology 81, no. 3 (2024): 562–576. 38710230 10.1016/j.jhep.2024.04.034

[39] J. D. Schumacher and G. L. Guo , “Regulation of Hepatic Stellate Cells and Fibrogenesis by Fibroblast Growth Factors,” BioMed Research International 2016 (2016): 8323747. 27699175 10.1155/2016/8323747 PMC5028827

[40] M. Noureddin , M. E. Rinella , N. P. Chalasani , et al., “Efruxifermin in Compensated Liver Cirrhosis Caused by MASH,” New England Journal of Medicine 392, no. 24 (2025): 2413–2424. 40341827 10.1056/NEJMoa2502242

[41] X.‐D. Zhou , V. W.‐S. Wong , and M.‐H. Zheng , “Resmetirom and GLP‐1 Agonists for MASH: Complementary Rather Than Exclusive,” npj Gut and Liver 1, no. 1 (2024): 4.

[42] A. Mangia and L. V. C. Valenti , “Safety Choice Drivers of the Coming Treatment Options for Non‐Cirrhotic Metabolic Steatohepatitis,” Liver International 45, no. 9 (2025): e70271. 40815181 10.1111/liv.70271

[43] M. Mallet , C. A. Silaghi , P. Sultanik , et al., “Current Challenges and Future Perspectives in Treating Patients With NAFLD‐Related Cirrhosis,” Hepatology 80, no. 5 (2024): 1270–1290. 37183906 10.1097/HEP.0000000000000456

[44] E. Brown , H. J. L. Heerspink , D. J. Cuthbertson , and J. P. H. Wilding , “SGLT2 Inhibitors and GLP‐1 Receptor Agonists: Established and Emerging Indications,” Lancet 398, no. 10296 (2021): 262–276. 34216571 10.1016/S0140-6736(21)00536-5

[45] N. Sattar , M. M. Y. Lee , S. L. Kristensen , et al., “Cardiovascular, Mortality, and Kidney Outcomes With GLP‐1 Receptor Agonists in Patients With Type 2 Diabetes: A Systematic Review and Meta‐Analysis of Randomised Trials,” Lancet Diabetes & Endocrinology 9, no. 10 (2021): 653–662. 34425083 10.1016/S2213-8587(21)00203-5

[46] F. Barkas , M. Elisaf , and H. Milionis , “Protection Against Stroke With Glucagon‐Like Peptide 1 Receptor Agonists: A Systematic Review and Meta‐Analysis,” European Journal of Neurology 26, no. 4 (2019): 559–565. 30629331 10.1111/ene.13905

[47] A. M. Lincoff , K. Brown‐Frandsen , H. M. Colhoun , et al., “Semaglutide and Cardiovascular Outcomes in Obesity Without Diabetes,” New England Journal of Medicine 389, no. 24 (2023): 2221–2232. 37952131 10.1056/NEJMoa2307563

[48] I. Lingvay , J. Deanfield , S. E. Kahn , et al., “Semaglutide and Cardiovascular Outcomes by Baseline HbA1c and Change in HbA1c in People With Overweight or Obesity but Without Diabetes in SELECT,” Diabetes Care 47, no. 8 (2024): 1360–1369. 38907684 10.2337/dc24-0764 PMC11282385

[49] M. Leung , S. M. MEYhöFER , B. Cariou , et al., “Semaglutide Improves Cardiovascular Outcomes in Patients With High Risk for Metabolic Dysfunction‐Associated Steatohepatitis—A Subgroup Analysis From the SELECT Trial,” Obesity Research & Clinical Practice 18, no. 5 (2024): S9.

[50] M. N. Kosiborod , J. Deanfield , R. Pratley , et al., “Semaglutide Versus Placebo in Patients With Heart Failure and Mildly Reduced or Preserved Ejection Fraction: A Pooled Analysis of the SELECT, FLOW, STEP‐HFpEF, and STEP‐HFpEF DM Randomised Trials,” Lancet 404, no. 10456 (2024): 949–961. 39222642 10.1016/S0140-6736(24)01643-X

[51] V. Perkovic , K. R. Tuttle , P. Rossing , et al., “Effects of Semaglutide on Chronic Kidney Disease in Patients With Type 2 Diabetes,” New England Journal of Medicine 391, no. 2 (2024): 109–121. 38785209 10.1056/NEJMoa2403347

[52] J. F. E. Mann , P. Rossing , G. Bakris , et al., “Effects of Semaglutide With and Without Concomitant SGLT2 Inhibitor Use in Participants With Type 2 Diabetes and Chronic Kidney Disease in the FLOW Trial,” Nature Medicine 30, no. 10 (2024): 2849–2856. 10.1038/s41591-024-03133-0 PMC11485243 38914124

[53] M. Marassi , A. Cignarella , G. T. Russo , et al., “Sex Differences in the Weight Response to GLP‐1RA in People With Type 2 Diabetes. A Long‐Term Longitudinal Real‐World Study,” Pharmacological Research 219 (2025): 107866. 40706779 10.1016/j.phrs.2025.107866

[54] K. G. Young , E. H. Mcinnes , R. J. Massey , et al., “Treatment Effect Heterogeneity Following Type 2 Diabetes Treatment With GLP1‐Receptor Agonists and SGLT2‐Inhibitors: A Systematic Review,” Communications Medicine 3, no. 1 (2023): 131. 37794166 10.1038/s43856-023-00359-w PMC10551026

[55] S. Romeo and L. Valenti , “Fifteen Years of PNPLA3: Transforming Hepatology Through Human Genetics,” Liver International 45, no. 9 (2025): e70240. 40747912 10.1111/liv.70240 PMC12315499

[56] E. Urias , N. R. Tedesco , A. Oliveri , et al., “PNPLA3 Risk Allele Association With ALT Response to Semaglutide Treatment,” Gastroenterology 166, no. 3 (2024): 515–517.e2. 37972824 10.1053/j.gastro.2023.11.018 PMC10922261

[57] O. Jamialahmadi , A. DE Vincentis , F. Tavaglione , et al., “Partitioned Polygenic Risk Scores Identify Distinct Types of Metabolic Dysfunction‐Associated Steatotic Liver Disease,” Nature Medicine 30, no. 12 (2024): 3614–3623. 10.1038/s41591-024-03284-0 PMC11645285 39653778

[58] M. Farokhnia , J. Tazare , C. L. Pince , et al., “Glucagon‐Like Peptide‐1 Receptor Agonists, but Not Dipeptidyl Peptidase‐4 Inhibitors, Reduce Alcohol Intake,” Journal of Clinical Investigation 135, no. 9 (2025): e188314. 40048376 10.1172/JCI188314 PMC12043080

[59] U.S. Food and Drug Administration , “FDA Approves First Treatment for Patients with Liver Scarring Due to Fatty Liver Disease,” (2025).

[60] E. Vilar‐Gomez , L. Calzadilla‐Bertot , V. Wai‐Sun Wong , et al., “Fibrosis Severity as a Determinant of Cause‐Specific Mortality in Patients With Advanced Nonalcoholic Fatty Liver Disease: A Multi‐National Cohort Study,” Gastroenterology 155, no. 2 (2018): 443–457.e17. 29733831 10.1053/j.gastro.2018.04.034

[61] R. Loomba , M. L. Hartman , E. J. Lawitz , et al., “Tirzepatide for Metabolic Dysfunction‐Associated Steatohepatitis With Liver Fibrosis,” New England Journal of Medicine 391, no. 4 (2024): 299–310. 38856224 10.1056/NEJMoa2401943

[62] G. Marchesini , R. Marzocchi , M. Noia , and G. Bianchi , “Branched‐Chain Amino Acid Supplementation in Patients With Liver Diseases,” Journal of Nutrition 135, no. 6 Suppl (2005): 1596S–1601S. 15930476 10.1093/jn/135.6.1596S

[63] A. Berzigotti , A. Albillos , C. Villanueva , et al., “Effects of an Intensive Lifestyle Intervention Program on Portal Hypertension in Patients With Cirrhosis and Obesity: The SportDiet Study,” Hepatology 65, no. 4 (2017): 1293–1305. 27997989 10.1002/hep.28992

[64] S. Pelusi and L. Valenti , “Hepatic Fat as Clinical Outcome and Therapeutic Target for Nonalcoholic Fatty Liver Disease,” Liver International 39, no. 2 (2019): 250–256. 30248234 10.1111/liv.13972

[65] V. L. Chen , T. R. Morgan , Y. Rotman , et al., “Resmetirom Therapy for Metabolic Dysfunction‐Associated Steatotic Liver Disease: October 2024 Updates to AASLD Practice Guidance,” Hepatology 81, no. 1 (2025): 312–320. 39422487 10.1097/HEP.0000000000001112

[66] European Association for the Study of the Liver , “EASL Clinical Practice Guidelines on Non‐Invasive Tests for Evaluation of Liver Disease Severity and Prognosis—2021 Update,” Journal of Hepatology 75, no. 3 (2021): 659–689. 34166721 10.1016/j.jhep.2021.05.025

[67] G. Pennisi , M. Enea , M. Romero‐Gomez , et al., “Risk of Liver‐Related Events in Metabolic Dysfunction‐Associated Steatohepatitis (MASH) Patients With Fibrosis: A Comparative Analysis of Various Risk Stratification Criteria,” Hepatology 79, no. 4 (2024): 912–925. 37796137 10.1097/HEP.0000000000000616

[68] D. Q. Huang , S. R. Sharpton , M. Amangurbanova , et al., “Clinical Utility of Combined MRI‐PDFF and ALT Response in Predicting Histologic Response in Nonalcoholic Fatty Liver Disease,” Clinical Gastroenterology and Hepatology 21, no. 10 (2023): 2682–2685.e4. 36075503 10.1016/j.cgh.2022.08.036

[69] H. Lin , H. W. Lee , T. C. Yip , et al., “Vibration‐Controlled Transient Elastography Scores to Predict Liver‐Related Events in Steatotic Liver Disease,” JAMA 331, no. 15 (2024): 1287–1297. 38512249 10.1001/jama.2024.1447 PMC10958386

[70] E. Vilar‐Gomez , Y. Martinez‐Perez , L. Calzadilla‐Bertot , et al., “Weight Loss Through Lifestyle Modification Significantly Reduces Features of Nonalcoholic Steatohepatitis,” Gastroenterology 149, no. 2 (2015): 367–378.e5; quiz e14–5. 25865049 10.1053/j.gastro.2015.04.005

[71] W. Wang , N. D. Volkow , N. A. Berger , P. B. Davis , D. C. Kaelber , and R. Xu , “Associations of Semaglutide With Incidence and Recurrence of Alcohol Use Disorder in Real‐World Population,” Nature Communications 15, no. 1 (2024): 4548. 10.1038/s41467-024-48780-6 PMC11133479 38806481

[72] C. S. Hendershot , M. P. Bremmer , M. B. Paladino , et al., “Once‐Weekly Semaglutide in Adults With Alcohol Use Disorder: A Randomized Clinical Trial,” JAMA Psychiatry 82, no. 4 (2025): 395–405. 39937469 10.1001/jamapsychiatry.2024.4789 PMC11822619

